# Delivery of CdiA Nuclease Toxins into Target Cells during Contact-Dependent Growth Inhibition

**DOI:** 10.1371/journal.pone.0057609

**Published:** 2013-02-28

**Authors:** Julia S. Webb, Kiel C. Nikolakakis, Julia L. E. Willett, Stephanie K. Aoki, Christopher S. Hayes, David A. Low

**Affiliations:** 1 Department of Molecular, Cellular and Developmental Biology, University of California Santa Barbara, Santa Barbara, California, United States of America; 2 Department of Chemistry and Biochemistry, University of California Santa Barbara, Santa Barbara, California, United States of America; 3 Biomolecular Science and Engineering Program, University of California Santa Barbara, Santa Barbara, California, United States of America; Arizona State University, United States of America

## Abstract

Bacterial contact-dependent growth inhibition (CDI) is mediated by the CdiB/CdiA family of two-partner secretion proteins. CDI systems deploy a variety of distinct toxins, which are contained within the polymorphic C-terminal region (CdiA-CT) of CdiA proteins. Several CdiA-CTs are nucleases, suggesting that the toxins are transported into the target cell cytoplasm to interact with their substrates. To analyze CdiA transfer to target bacteria, we used the CDI system of uropathogenic *Escherichia coli* 536 (UPEC536) as a model. Antibodies recognizing the amino- and carboxyl-termini of CdiA^UPEC536^ were used to visualize transfer of CdiA from CDI^UPEC536+^ inhibitor cells to target cells using fluorescence microscopy. The results indicate that the entire CdiA^UPEC536^ protein is deposited onto the surface of target bacteria. CdiA^UPEC536^ transfer to *bamA101* mutants is reduced, consistent with low expression of the CDI receptor BamA on these cells. Notably, our results indicate that the C-terminal CdiA-CT toxin region of CdiA^UPEC536^ is translocated into target cells, but the N-terminal region remains at the cell surface based on protease sensitivity. These results suggest that the CdiA-CT toxin domain is cleaved from CdiA^UPEC536^ prior to translocation. Delivery of a heterologous *Dickeya dadantii* CdiA-CT toxin, which has DNase activity, was also visualized. Following incubation with CDI^+^ inhibitor cells targets became anucleate, showing that the *D.dadantii* CdiA-CT was delivered intracellularly. Together, these results demonstrate that diverse CDI toxins are efficiently translocated across target cell envelopes.

## Introduction

Contact-dependent growth inhibition (CDI) is a mechanism that allows Gram-negative bacteria to inhibit the growth of neighboring bacteria upon direct cell-to-cell contact [1]–[3]. CDI is mediated by the CdiB/CdiA family of two-partner secretion proteins, which are found in many Gram-negative bacteria including α, β, and γ-proteobacteria. CdiB is a predicted β-barrel protein required for export of the CdiA effector protein across the outer membrane. CdiA exoproteins are exceptionally large (200–600 kDa) and are thought to form β-helical filaments several hundred angstroms in length based on their homology to filamentous hemagglutinin (FHA) [4]. The current model of CDI postulates that CdiA filaments extend like quills from inhibitor cells, allowing the effector protein to engage specific receptors on the surface of target bacteria. Upon cell-cell contact, a toxin derived from CdiA is delivered into the target cell to inhibit growth. Analysis of CdiA proteins from several different bacteria has shown that the growth inhibitory activity is localized to the extreme C-terminus [1], [5], [6], and this region has been collectively termed “CdiA-CT”. Remarkably, the CdiA-CT region is highly variable between CDI systems, indicating that inhibitor bacteria deploy a variety of distinct toxins. The CdiA-CT from *Escherichia coli* EC93 appears to form pores in the inner membrane because this toxin dissipates the proton motive force [7]. Other characterized CdiA-CTs have toxic nuclease activities. The CdiA-CT from uropathogenic *E. coli* 536 (UPEC 536) is a tRNA anticodon nuclease [8], and the toxin from the plant pathogen *Dickeya dadantii* 3937 has avid DNase activity when analyzed *in vitro* [1]. Because CDI systems target bacteria, CDI^+^ inhibitor cells must also produce an immunity protein (CdiI) to protect themselves from autoinhibition [2]. CdiI immunity proteins are also highly variable and specifically bind their cognate CdiA-CT to block toxin activity, but they provide no protection from the CDI toxins deployed by other bacteria [1], [5]. Thus, CDI systems form a network of toxin/immunity proteins that function in bacterial growth competition.

Because CDI systems inhibit growth by either degrading nucleic acids or disrupting the bacterial inner membrane, CdiA-CT toxins must be delivered across the target cell envelope. The mechanisms of toxin translocation remain unknown, but genetic approaches have identified two envelope proteins that may facilitate the process for the *E. coli* EC93 CDI system. Selections for CDI^EC93^ resistant *E. coli* mutants led to the isolation of a single transposon insertion in the promoter region of *bamA* (*bamA101*) and several different disruptions of the *acrB* coding sequence [9]. BamA is an essential outer membrane protein that forms the core of the β-barrel assembly complex in Gram-negative bacteria [10]–[14]. The *bamA101* mutation decreases BamA surface expression approximately five-fold and confers partial resistance to EC93 inhibitor cells [9]. CDI^+^ inhibitor cells bind to *bamA101* mutants less efficiently than wild-type *bamA^+^* cells and *E. coli* target cells are protected from CDI by pre-incubation with anti-BamA antibodies [9]. Together, these observations argue that BamA is the CDI receptor in *E. coli* and suggest that it may also serve as a conduit for CdiA-CT transport across the outer membrane. AcrB is a trimeric inner membrane protein that functions with AcrA and TolC as a multi-drug efflux pump [15], [16]. TolC is a periplasm-spanning porin that is parasitized to translocate a subset of colicin toxins [17]. However, deletion of either *acrA* or *tolC* genes has no effect on CDI^EC93^ resistance [9], indicating that the role of AcrB in CDI^EC93^ resistance is distinct from its efflux function and the colicin import pathway. AcrB is also required for growth inhibition when the isolated CdiA-CT^EC93^ domain is produced inside *E. coli* cells [7]. This latter finding suggests that AcrB facilitates toxin insertion into the inner membrane from both periplasmic and cytoplasmic compartments. Such a function implies that AcrB is specific to the EC93 CDI pathway, and indeed *acrB* mutants are not resistant to CDI mediated by UPEC 536. Based on these observations, it appears that the various CdiA-CT toxins could each exploit distinct pathways to enter target cells.

Here, we use cell biological approaches to visualize CDI toxin delivery into target bacteria. Immunofluorescence microscopy using antibodies to antigens at the N- and C-termini of CdiA^UPEC536^ suggests that the entire CdiA protein is transferred to the surface of target cells during growth inhibition. CdiA transfer is proportional to the level of BamA receptor on target cells. CdiA-CT^UPEC536^ antigen is detected in the cytoplasm of target cells, suggesting that the toxin is cleaved from full-length CdiA prior to translocation of the CdiA-CT. Furthermore, we demonstrate the translocation of a heterologous CDI toxin from *Dickeya dadantii* 3937 into *E. coli* target cells. We replaced the CdiA-CT of CdiA^EC93^ with the CdiA-CT^3937−2^ DNase domain to create a chimeric effector protein, and monitored toxin delivery by staining target cell DNA with DAPI. Target bacteria lost DAPI staining in response to co-culture with chimeric CDI^3937−2^ inhibitor cells, consistent with the complete degradation of their genomes. Together, these results demonstrate that diverse CDI toxins are efficiently translocated into *E. coli* cells.

## Results

### Detection of CdiA on the Surface of Inhibitor Cells

**Figure 1:**
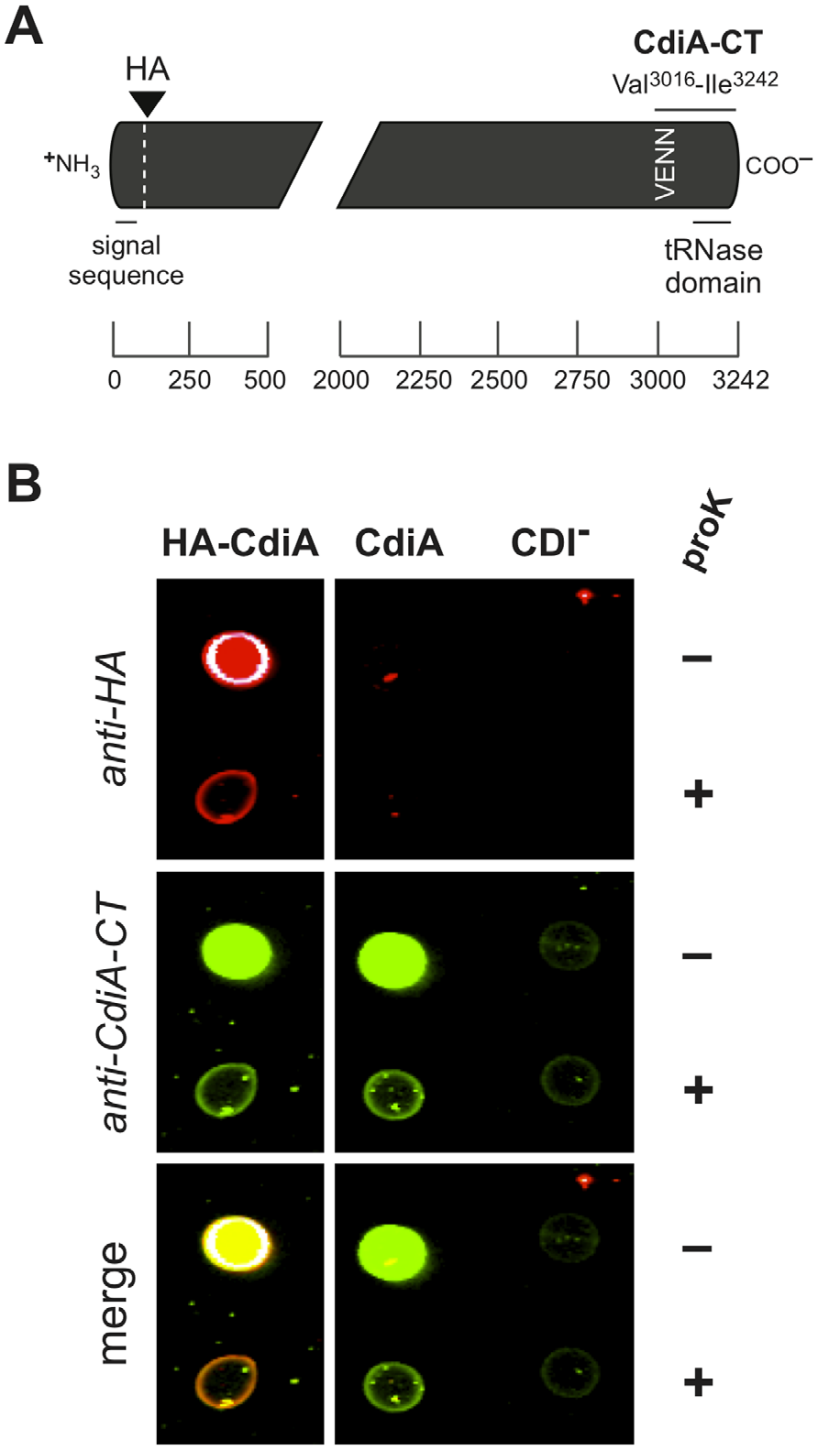
CdiA^UPEC536^ and HA-CdiA^UPEC536^ are exported to the cell surface. A) Schematic of the CdiA^UPEC536^ exoprotein depicting the locations of the inserted N-terminal hemagglutinin (HA) epitope tag and the CdiA-CT region (residues Val3016– Ile3242) used to generate anti-CdiA-CT^UPEC536^ polyclonal antibodies. Regions corresponding to the secretion signal sequence and the toxic tRNase domain are indicated. The vertical VENN sequence demarcates the N-terminal margin of the variable CdiA-CT sequence. Residue numbers are shown by the scale bar below. B) Whole-cell immunoblot analysis of *E. coli* cells expressing CdiA^UPEC536^ and HA-CdiA^UPEC536^. Cells expressing HA-CdiA^UPEC536^ (HA-CdiA), CdiA^UPEC536^ (CdiA) or no effector protein (CDI^-^) were fixed without permeabilization and stained with anti-HA or anti-CdiA-CT^UPEC536^ (anti-CdiA-CT) antibodies. Where indicated, cells were treated with proteinase K (proK) prior to fixation. Stained cells were spotted onto nitrocellulose membrane and analyzed with an Odyssey infrared imager.

We chose the CDI system from uropathogenic *Escherichia coli* 536 (UPEC 536) [18] to examine toxin delivery because CdiA-CT^UPEC536^ is a tRNase with substrates in the cytosol of target cells. To facilitate genetic manipulations, we used a cosmid-borne copy of the UPEC 536 *cdiBAI* gene cluster, which is sufficient to confer the inhibitor cell phenotype to CDI^-^ strains of *E. coli* K-12 [8]. We introduced a hemagglutinin (HA) peptide epitope near the mature N-terminus of CdiA^UPEC536^ for detection by immunofluorescence (Fig. 1A). Additionally, we raised polyclonal antiserum against the CdiA-CT^UPEC536^ toxin region. Thus, the N- and C-termini of CdiA^UPEC536^ can be tracked simultaneously using the appropriate fluorescently labeled antibodies. Because the HA epitope was inserted adjacent to the signal sequence (Fig. 1A), we first examined surface expression of HA-CdiA^UPEC536^ by whole-cell immunoblot to confirm that the modified exoprotein is still secreted. Cells were incubated with fluorescent anti-HA antibodies under conditions that do not permeabilize the outer membrane based on staining for a periplasmic marker and then blotted onto a nitrocellulose membrane (Fig. S1A). A strong fluorescent signal was detected from cells expressing HA-CdiA^UPEC536^, but only background fluorescence was observed from cells expressing untagged CdiA^UPEC536^ or control CDI^-^ cells carrying an empty cosmid vector (Fig. 1B). The HA signal was sensitive to treatment with proteinase K (Fig. 1B), consistent with epitope exposure on the cell surface. However, to conclusively determine if the immunoblot procedure only detects cell-surface antigens, we repeated the analysis using antibodies to maltose-binding protein (MBP), which is localized to the periplasm [19]. MBP was not detected under the original immunoblot conditions, but a strong signal was obtained when the cells were permeabilized with Triton X-100 (Fig. S1A). Notably, the MBP signal was insensitive to proteinase K, indicating that periplasmic and presumably cytoplasmic antigens are protected from the protease (Fig. S1A). Thus, fixation conditions can be controlled to differentiate between surface exposed and internal antigens. We repeated this analysis using polyclonal antibodies to CdiA-CT^UPEC536^ and found that the toxin domain was present on the surface of inhibitor cells expressing either HA-tagged or untagged CdiA^UPEC536^ (Fig. 1B).

### CdiA^UPEC536^ is Delivered onto the Surface of Target Cells

**Figure 2:**
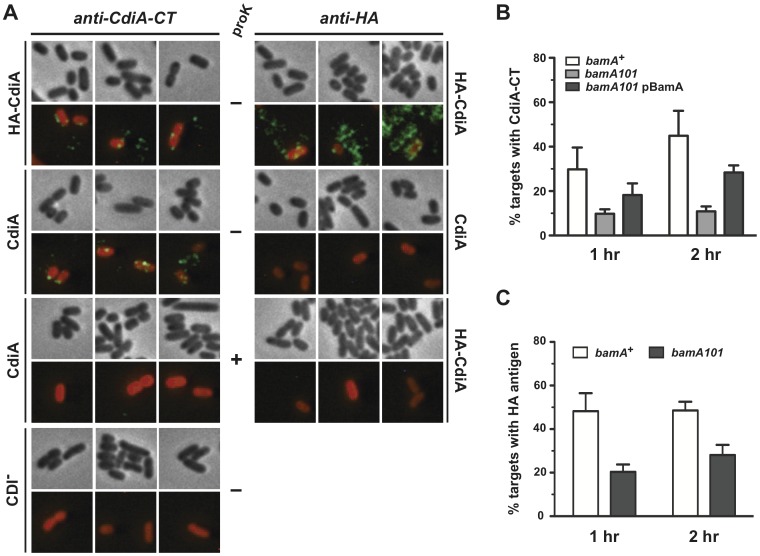
CdiA^UPEC536^ is transferred to the surface of target cells. A) Immunofluorescence microscopy of CDI^UPEC536^ co-cultures. Non-fluorescent inhibitor cells (expressing either CdiA^UPEC536^ or HA-CdiA^UPEC536^) were mixed with red fluorescent target cells (2∶1 inhibitor-to-target ratio) for 1 h, then analyzed by fluorescence microscopy using anti-CdiA-CT^UPEC536^ (anti-CdiA-CT) or anti-HA antibodies as described in Methods. Green fluorescence is indicative of anti-CdiA-CT and anti-HA staining of cell surfaces. Inhibitor cells in the CDI^-^ panel carry an empty vector without a CDI system. Where indicated (+), samples were treated with proteinase K prior to fixation, but cells were not permeabilized. B) Transfer of CdiA-CT^UPEC536^ antigen to target cells. The percentage of *bamA* ^+^ and *bamA101* target cells with CdiA-CT^UPEC536^ antigen was quantified after one and two hours of co-culture with CdiA^UPEC536^ inhibitor cells. The *bamA101* allele was complemented with plasmid pBamA (corresponding to pDAL950 in Table 1). C) Transfer of HA antigen to target cells. Quantifications in panels B and C were determined by analysis of 150 target cells from two independent experiments. The reported values represent the average ± SEM.

Having established that HA-CdiA^UPEC536^ is secreted, we next tested its inhibition activity in growth competition assays. Inhibitor cells expressing HA-CdiA^UPEC536^ were incubated with target *E. coli* cells in shaking broth cultures, and the number of viable target cells were quantified. After four hours of co-culture with HA-CdiA^UPEC536^ inhibitors, target cell counts decreased ∼100-fold, whereas CDI^-^ mock inhibitor cells carrying the cosmid vector did not inhibit target cell growth (Fig. S2). Moreover, HA-CdiA^UPEC536^ inhibited growth to the same extent as wild-type CdiA^UPEC536^ (Fig. S2), demonstrating that the HA-tagged effector protein is fully functional in CDI. We examined the co-cultures with fluorescence microscopy to determine if CdiA-associated antigens are transferred to target cells during CDI. In these experiments, target cells were labeled with the red fluorescent protein DsRed to differentiate them from unlabeled CDI^+^ inhibitor cells. Cell surfaces were stained with fluorescein-labeled antibodies to CdiA-CT^UPEC536^, which revealed toxin antigen on many cells in the co-culture including a large proportion of target cells (Fig. 2A). CdiA-CT^UPEC36^ immunostaining was specific with virtually no signal detected in co-cultures containing CDI^-^ mock inhibitor cells (Fig. 2A). All of the CdiA-CT^UPEC536^ antigen could be removed with proteinase K treatment prior to antibody staining (Fig. 2A), indicating that the toxin domain is exposed on the surface of target cells. Co-cultures containing HA-CdiA^UPEC536^ inhibitor cells also showed similar transfer of CdiA-CT^UPEC536^ toxin antigen onto target bacteria (Fig. 2A). Somewhat unexpectedly, immunostaining for the N-terminal HA epitope revealed that this antigen is also delivered to the surface of target cells (Fig. 2A). HA epitope staining was specific with no signal detected in co-cultures containing cells that express untagged CdiA^UPEC536^ (Fig. 2A). Together, these experiments demonstrate that both the N-terminal and C-terminal regions of CdiA^UPEC535^ are transferred onto the surface of target cells.

Our previous studies have shown that CdiA fragments are released from inhibitor cells [2]. Soluble CdiA fragments have no growth inhibition activity but could bind to cells non-specifically and account for the fluorescent signal observed on target bacteria. Therefore, we tested whether CdiA^UPEC536^ delivery is influenced by the level of BamA receptor on target cells, reasoning that only BamA-dependent transfer is relevant to the CDI pathway. Co-culture and immunofluorescence experiments were repeated with target cells that carry the *bamA101* mutation, which reduces BamA surface expression approximately five-fold [9]. Quantification of *bamA101* target cells with HA and CdiA-CT^UPEC536^ antigens on their surface showed that CdiA^UPEC536^ delivery was reduced two- to three-fold compared to *bamA* ^+^ target cells (Figs. 2B & 2C). Complementation of *bamA101* cells with a plasmid expressing BamA doubled the number of cells with CdiA-CT^UPEC536^ surface staining, confirming that reduced CdiA^UPEC536^ delivery is due to low BamA levels. Together, these data indicate that delivery of CdiA^UPEC536^ to the surface of target cells is BamA-dependent.

### CdiA-CT^UPEC536^ is Translocated into the Cytoplasm of Target Cells during CDI

**Figure 3:**
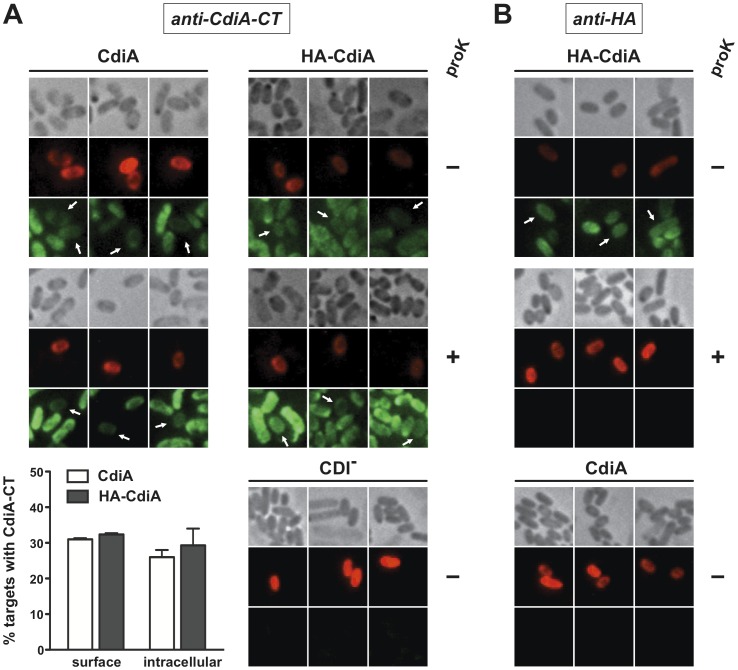
CdiA-CT^UPEC536^ is delivered into target cells. A) Anti-CdiA-CT^UPEC536^ immunofluorescence microscopy of CDI^UPEC536^ co-cultures. Non-fluorescent inhibitor cells expressing either CdiA^UPEC536^ (CdiA) or HA-CdiA^UPEC536^ (HA-CdiA) were mixed with red fluorescent target cells (2∶1 inhibitor-to-target ratio) for 1 h. Cells were then fixed and permeabilized for fluorescence microscopy using anti-CdiA-CT^UPEC536^ (anti-CdiA-CT) antibodies as described in Methods. Green fluorescence is indicative of anti-CdiA-CT immunostaining. Where indicated (+), cells were treated with proteinase K prior to fixation. The histogram quantifies the percentage of target cells (average ± SEM) with surface and internal CdiA-CT^UPEC536^ antigen staining. At least 150 target cells from two independent experiments were scored for the quantification of CdiA delivery. B) Anti-HA epitope immunofluorescence microscopy of CDI^UPEC536^ co-cultures. Co-culture conditions and sample preparation was as described in panel A except that anti-HA antibodies were used for immunofluorescence. Green fluorescence is indicative of anti-HA immunostaining. Where indicated (+), cells were treated with proteinase K prior to fixation.

We next sought to detect CdiA-derived antigens inside target bacteria. Control experiments using antibodies to periplasmic MBP showed that internal antigens are only detectable when cells are permeabilized with EDTA and lysozyme treatment (Fig. S1B). We applied the EDTA/lysozyme procedure to CDI co-cultures and observed diffuse CdiA-CT^UPEC536^ staining of both inhibitor and target cells (Fig. 3A). This staining was specific with no signal detected from either CDI^-^ cells using anti-CdiA-CT antibodies (Fig. 3A) or CdiA^+^ cells using anti-HA antibodies (Fig. 3B). Because there is significant CdiA-CT^UPEC536^ antigen on cell surfaces (see Fig. 2A), the signal from permeabilized cells likely represents the sum of both surface and internal antigens. Therefore, to specifically visualize internal CdiA-CT^UPEC536^, we removed surface antigen with proteinase K treatment and then permeabilized the cells for immunostaining. Using the protease/permeabilization protocol, we found that virtually all inhibitor cells and approximately 30% of the target cells contain internal CdiA-CT^UPEC536^ antigen (Fig. 3A). The HA-CdiA^UPEC536^ effector protein delivered CdiA-CT^UPEC536^ antigen to targets at a level similar to untagged CdiA^UPEC536^ (Fig. 3A), indicating that the N-terminal HA tag does not adversely affect CdiA-CT^UPEC536^ translocation. We used the same fixation regimen to determine whether the N-terminal HA epitope is also internalized by target cells. Although there was abundant HA epitope detected on cells in the co-culture, this signal was ablated by proteinase K treatment (Fig. 3B), strongly suggesting that all of the HA peptide is surface exposed. These data indicate that the N-terminal HA epitope remains on the target cell surface, whereas the CdiA-CT^UPEC536^ toxin is internalized.

### CdiA-CT Translocation into Target Cells is a General Feature of CDI

**Figure 4:**
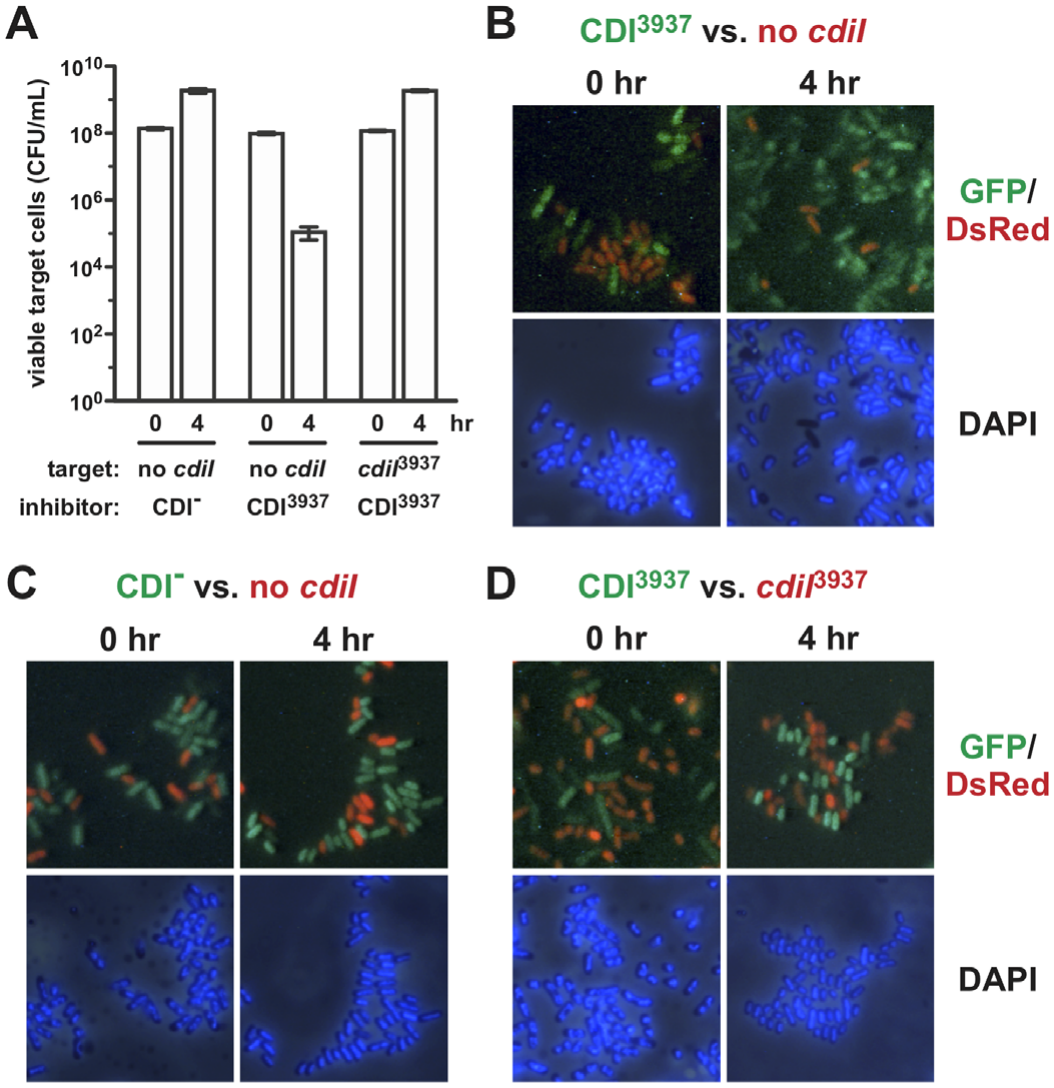
The CdiA-CT^3937^ ^−2^ DNase is delivered into *E. coli* cells. A) The chimeric EC93-Dd3937 CDI system is functional. Mock inhibitors (CDI^-^) or CDI^3937^ cells were co-cultured at a 1∶1 ratio with target bacteria that carry plasmid pBR322 (*cdiI* ^-^) or pDAL852 (*cdiI* ^3937−2^). Co-cultures were sampled at the indicated times and viable target cells quantified as colony forming units (CFU) per mL. Reported values are the average ± SEM for three independent experiments. For panels B, C and D, inhibitor cells were labeled with GFP (green) and targets with DsRed (red) to differentiate the two cell populations by fluorescence microscopy. Competitions were conducted as described for panel A, and cells were stained with DAPI to visualize DNA. B) Inhibitor cells expressing CdiA-CT^3937−2^ were co-cultured with *cdiI^-^* targets, C) Mock inhibitors (CDI^-^) were co-cultured with *cdiI^-^* targets. D) Inhibitors expressing CDI^3937^ were co-cultured with targets expressing CdiI^3937−2^ immunity.

**Figure 5:**
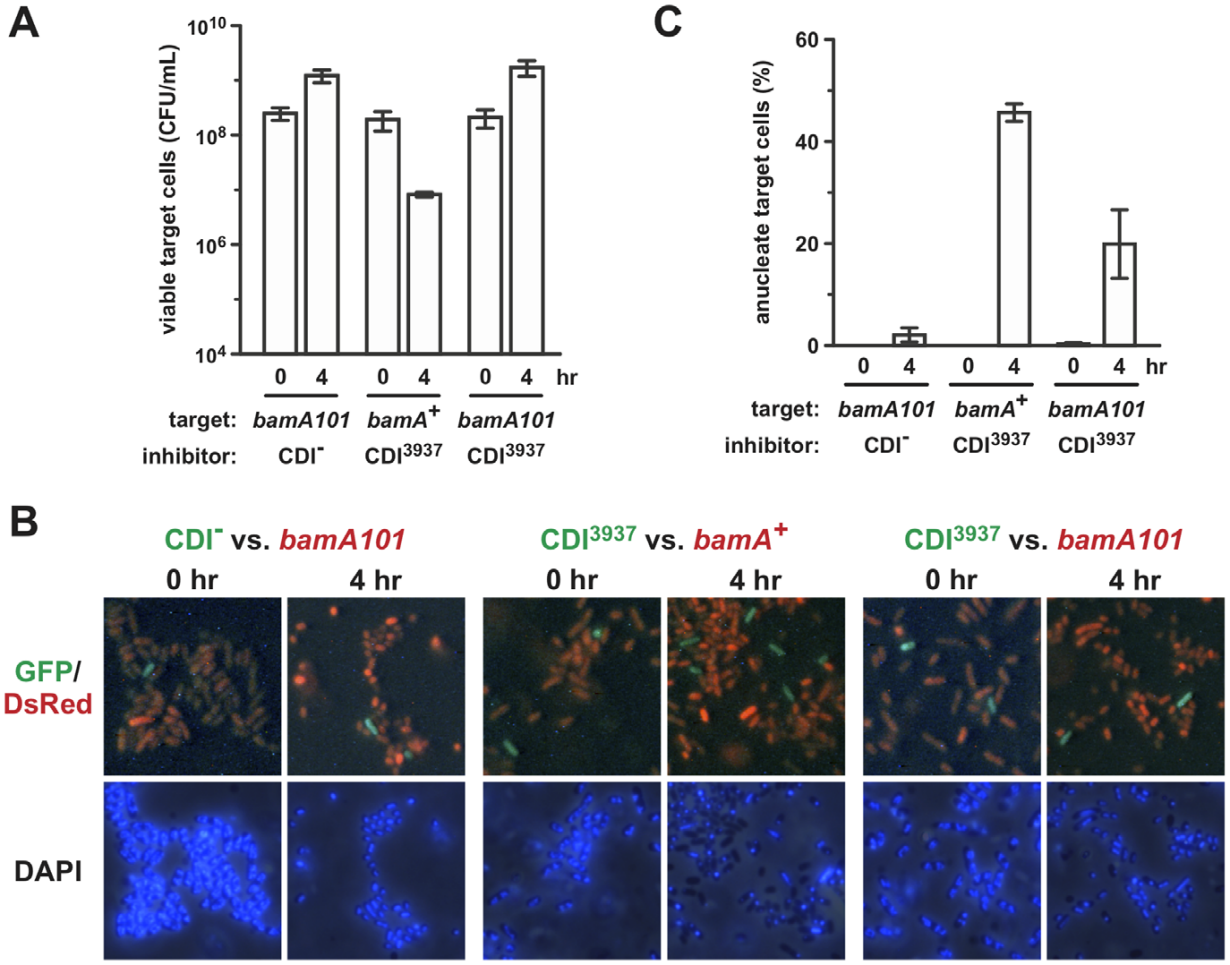
DNase delivery is dependent upon the BamA receptor. A) *bamA101* target cells are resistant to the chimeric EC93-Dd3937 CDI system. Mock inhibitors (CDI^-^) or CDI^3937^ cells were co-cultured with *bamA* ^+^ cells or *bamA101* cells at a 1∶100 inhibitor to target ratio. Cultures were sampled at the indicated times and viable target cells quantified as colony forming units (CFU) per mL. Reported values are the average ± SEM for three independent experiments. B) Visualization of DNase delivery. Competitions were conducted as described for panel A, but inhibitors were labeled with GFP (green) and targets with DsRed (red) to differentiate the two cell populations by fluorescence microscopy. Cells were stained with DAPI to visualize DNA. C) Quantification of anucleate target cells. The percentage of anucleate target cells (defined as cells lacking DAPI staining) was determined by inspection of random microscopy fields. Two independent co-cultures were analyzed and between 186–260 target cells were scored for each competition. Values represent the average ± SEM.

CDI systems deploy a variety of toxins with diverse sequences, all of which must be efficiently transported into target bacteria. Therefore, we sought to visualize the delivery of another distinct CDI toxin into *E. coli* cells. We showed previously that the CdiA-CT^3937−2^ toxin from *Dickeya dadantii* 3937 is a non-specific DNase that degrades plasmid DNA *in vitro* [1]. We reasoned that this toxin should alter target cell DNA, which could be visualized by DAPI staining and microscopy. The CDI system from *D. dadantii* 3937 does not target *E. coli* [1]. Therefore, we replaced the CdiA-CT of *E. coli* CdiA^EC93^ with the CdiA-CT^3937−2^ toxin to generate a chimeric effector protein capable of delivering the DNase into *E. coli* cells. We first tested the CdiA^EC93^-CT^3937−2^ chimera to determine whether it is active in CDI. CDI^3937−2^ inhibitor cells were mixed with target cells at a 1∶1 ratio and incubated in shaking broth cultures. After four hours of co-culture, the number of viable target cells decreased ∼1,000-fold (Fig. 4A). In contrast, target cells carrying a plasmid-borne copy of the *cdiI* ^3937−2^ immunity gene grew unimpeded (Fig. 4A), indicating that the grafted CdiA-CT^3937−2^ toxin is responsible for growth inhibition. To visualize DNase activity in target cells, we repeated the co-culture experiments with GFP labeled inhibitor cells and DsRed labeled targets and stained the cells with DAPI to visualize nucleoids. Upon initial mixing, inhibitor and target cells both exhibited similar nucleoid staining and morphology (Fig. 4B). However, a fraction of target cells lost DAPI staining after four hours of co-culture with CDI^3937−2^ inhibitor cells (Fig. 4B), consistent with the degradation of genomic DNA. This loss of DAPI staining was CDI-dependent (Fig. 4C), and target cells that express the *cdiI* ^3937−2^ immunity gene showed no changes in nucleoid appearance during co-culture with inhibitors (Fig. 4D). Finally, we tested whether DNase delivery was dependent upon BamA. We repeated the growth competition experiments using an inhibitor to target cell ratio of 1∶100 and found that *bamA101* targets were resistant to the chimeric CDI^3937−2^ system under these conditions (Fig. 5A). Microscopic analysis showed that wild-type *bamA^+^* cells were more likely to lose cellular DNA (∼45% anucleate cells) compared to *bamA101* targets (∼20% anucleate cells) after co-culture with CDI^3937−2^ inhibitors (Figs. 5B & 5C). Together, these results indicate that CdiA-CT^3937−2^ is translocated into *E. coli* in a BamA-dependent manner and that the toxin destroys target cell genomic DNA to inhibit growth.

## Discussion

The experiments presented here represent the first examination of CdiA effector protein transfer between bacteria during contact-dependent growth inhibition. We find that both the N-terminal and C-terminal regions of CdiA are deposited onto the surface of target cells. Although it is formally possible that only N- and C-terminal CdiA fragments are transferred, the delivery of both antigens is dependent upon BamA expression, strongly suggesting that the entire effector protein is transferred to the surface of target bacteria. We find that CdiA-CTs are translocated into target cells, presumably into the cytoplasm where the nucleic acid target molecules reside. In contrast, we did not detect N-terminal HA epitope inside target bacteria, indicating that the N-terminus of CdiA is not efficiently internalized. Presumably, some portion of the CdiA-CT is cleaved prior to translocation, but it is still unclear how much of the CdiA protein is transported into the target cell cytoplasm. Biochemical analysis indicates that the tRNase activity of CdiA^UPEC536^ is contained within the C-terminal 145 residues, and this fragment is sufficient to inhibit growth when expressed inside *E. coli* cells [8]. These observations indicate that at a minimum the extreme C-terminal tRNase domain must be translocated into target cells during CDI. We note that the CdiA-CT^UPEC536^ region appears to be comprised of two functional domains, the C-terminal tRNase domain and an N-terminal region of unknown function [8]. We suspect that the N-terminal region that immediately follows the VENN motif facilitates translocation of the C-terminal tRNase domain through the target cell envelope, but this hypothesis remains to be rigorously tested.

CdiA and CdiB are members of the two-partner secretion (TPS) protein family, which together with the related autotransporter systems constitute the type V secretion systems of Gram-negative bacteria. TPS systems encode two proteins: TpsB partners are outer membrane β-barrel proteins and the secreted TpsA partners are large, hemagglutinin-repeat containing exoproteins. [20] The filamentous hemagglutinin (FHA)/FhaC proteins from *Bordetella* species and the HMW1/2 proteins of *Haemophilus influenzae* serve as paradigms for TPS protein export and assembly onto the outer membrane. Although the FHA and HMW1 TpsA proteins are related to CdiA, they do not appear to contain toxic effector domains and instead function as adhesins. Both FHA and HMW1 adhesins are held in the outer membrane through non-covalent associations with their TpsB protein partner. However, the orientation of TpsA protein appears to be variable. Based on epitope accessibility studies, FHA is predicted to interact with FhaC through its N-terminus, thus projecting the C-terminus away from the cell surface [21]. In contrast, HMW1 is assembled in the opposite orientation, allowing the N-terminal adhesin domain to be projected outward [22], [23]. Because CdiA is transferred between bacteria, the experiments described here are unable to unambiguously determine the orientation of CdiA on the inhibitor cell surface. The entire CdiA molecule appears to be transferred to target cells, and thus either orientation should be feasible, as long as the BamA-binding region of CdiA is accessible to its ligand. The BamA-binding domain of CdiA is unknown, but is presumably N-terminal to the variable CdiA-CT toxin region. Localization of the BamA-binding region on CdiA is one approach that may suggest a model for exoprotein topology in the outer membrane of inhibitor cells. Alternatively, antibody accessibility studies similar to those performed with FHA should be informative if the transfer of CdiA between inhibitor cells could be prevented.

CDI systems deploy toxins that are similar in activity to colicins and other bacteriocins. Colicins are soluble protein toxins that specifically target *E. coli* and a few closely related enterobacteria [17]. In general, colicins have a conserved architecture comprised of an N-terminal translocation domain, a central receptor-binding domain, and a C-terminal toxin domain [24]. The receptor-binding domain interacts with specific β-barrel proteins on the surface of susceptible bacteria, and then the translocation domain exploits either the Tol or Ton import systems to cross the outer membrane. The receptor-binding domain is thought to remain at the cell surface while the toxin domain is processed and released into the cytoplasm [25], [26]. A similar process probably occurs during CDI, with only the C-terminal region of CdiA transported into target bacteria. However, CdiA exoproteins are clearly distinct from colicins and do not rely on the Tol or Ton systems for import [2], [9]. Perhaps the most striking difference is that CDI requires direct cell-to-cell contact to deliver toxins – soluble CdiA fragments containing the CdiA-CT region have no effect on cell growth [2]. The requirement for cell-cell contact is not understood and is somewhat perplexing given that the entire CdiA molecule is transferred to the surface of target cells. Perhaps the entire CdiA molecule is required for toxin translocation. The N-terminus of CdiA may contain a domain(s) necessary for toxin translocation into target cells. This could include the CdiA cleavage site or a protease domain that is required for protein processing. It is remarkable that multiple pathways exist for the import of relatively large toxin domains through the Gram-negative cell envelope. We are currently identifying additional genes required for CDI, which will likely inform mechanistic models of CDI toxin import.

## Materials and Methods

### Plasmids and Bacterial Strains

All plasmids and *E. coli* strains used in this study are listed in Table 1. Bacteria were grown in either LB medium (1% NaCl, 1% tryptone, 0.5% yeast extract) or tryptone broth (TB) (1% tryptone, 0.5% NaCl) supplemented with 10 mM MgSO_4_. Antibiotics were used at the following concentrations unless otherwise indicated: ampicillin (Amp), 100 µg/mL; chloramphenicol (Cm), 34 µg/mL; kanamycin (Kan), 50 µg/mL; streptomycin (Str), 50 µg/mL; and rifampicin (Rif), 200 µg/mL.

**Table 1:** Bacterial strains and plasmids used in this study.

Strain or plasmid	Genotype or description^a^	Reference/Source
***Strains***		
EPI100	F^-^ *mcrA* Δ*(mrr-hsdRMS-mcrBC) φ80dlacZ*Δ*M15* Δ*lacX74 recA1*	Epicentre
	*endA1 araD139* Δ*(ara, leu)7697 galU galK* - *rpsL nupG*, Str^R^	
DY378	W3110 λ*cI857* Δ*(cro-bioA*)	[30]
MC4100	F^-^ *araD139* Δ*(argF-lac)U169 rpsL150 relA1 flbB5301 deoC1*	[35]
	*ptsF25 rbsR*, Str^R^	
JCM158	F^-^ Δ*(argF-lac)U169 rpsL150 relA1 flbB5301 deoC1 ptsF25*	[36]
	*rbsR*, Str^R^	
TT23216	*Salmonella enterica* strain containing chloramphenicol	John Roth
	acetyltransferase gene (*cat*), Cm^R^	
UPEC 536	Human uropathogenic *E. coli* isolate	[37]
JW3994	F^-^ Δ*(araD-araB)567* Δ*lacZ4787*(::*rrnB-3*) *rph-1* Δ*(rhaD-*	[38]
	*rhaB)568* Δ*malE730::kan hsdR514*, Kan^R^	
DL4259	MC4100 λ640-13 P*_papIB_*-*gfp-mut3*	This study
DL5562	JCM158 Δ*wzb::kan*, Kan^R^	[9]
DL5564	JCM158 Δ*wzb*	[9]
DL5622	JCM158 Δ*wzb bamA101*::*kan*, Kan^R^	[9]
DL6105	*E. coli* EC93 Δ*cdiA-CT/*Δ*cdiI*	[5]
CH2016	X90 (DE3) Δ*rna* Δ*slyD::kan*, Kan^R^	[33]
CH8251	MC4100 *rif* ^r^ (spontaneous rifampicin-resistance), Str^R^ Rif^R^	This study
CH9122	MC4100 *rif* ^r^ Δ*wzb bamA101::kan*, Str^R^ Rif^R^ Kan^R^	This study
CH9634	MC4100 *rif* ^r^ Δ*wzb::kan*, Str^R^ Rif^R^ Kan^R^	This study
***Plasmids***		
pWEB-TNC	Cosmid cloning vector, Cm^R^ Amp^R^	Epicentre
pDsRed-Express2	pUC19 derivative encoding DsRed, Amp^R^	Clontech
pBR322	cloning vector, Amp^R^ Tet^R^	[39]
pCH70	pBluescript with *kan* cassette, Amp^R^, Kan^R^	[28]
pKSS	pBluescript with pheS* counter-selectable marker, Amp^R^	[29]
pKSS-Kan	pKSS with *kan* cassette from pCH70, Amp^R^, Kan^R^	This study
pZS21-*bamA*	pZS21 expressing BamA, Kan^R^	[40]
pDAL660Δ1-39	pWEB-TNC containing 16,734 bp *cdiBAI* sequence from	[2]
	*E. coli* EC93	
pDAL672	p*lac*-DsRed, Amp^R^	[2]
pDAL852	pBR322 expressing the *cdiI* ^3937−2^ immunity gene, Amp^R^	[1]
pDAL866	pWEB-TNC::*cdiBAI* ^UPEC536^, Cm^R^ Amp^R^	[8]
pDAL878	pDAL660Δ1-39 with deletion of *cdiA-CT* ^EC93^ */cdiI* ^EC93^ region	This study
	(CDI^-^), Cm^R^	
pDAL879	Cosmid expressing chimeric CdiA^EC93^-CT_o1_^EC93^ protein, Cm^R^	[5]
pDAL903	pWEB-TNC::*cdiBA* ^HA^ *I* ^UPEC536^; encodes HA-CdiA^UPEC536^ effector	This study
	protein, Cm^R^ Amp^R^	
pDAL950	pZS21-*bamA* with chloramphenicol resistance cassette, Cm^R^	This study
pCH9817	pTrc-DsRed, Amp^R^	This study
pCH10323	Cosmid expressing chimeric CdiA^EC93^-CT^3937−2^ protein, Cm^R^	This study
pCH10412	pKSS-Kan with *cdiA* ^EC93^ homology regions cloned upstream	This study
	and downstream of kan-pheS*, Amp^R^, Kan^R^	

a Abbreviations: Amp^R^, ampicillin resistant; Cm^R^, chloramphenicol resistant; Kan^R^, kanamycin resistant; Rif^R^, rifampicin resistant; Str^R^, streptomycin resistant.

Cosmid pDAL866 constitutively expresses the *cdiBAI* gene cluster from UPEC 536 [8]. The HA epitope coding sequence was introduced between the codons for Ala32 and Val33 of UPEC 536 *cdiA* using overlapping extension PCR (OE-PCR) [27]. Fragments of *cdiA* ^UPEC536^ were amplified using oligonucleotide pairs 2051/2064 and 2065/2052, and the two PCR products were joined by a second amplification using primers 2051/2052 (Table S1). The resulting product was digested with NsiI and StuI restriction endonucleases and ligated to NsiI/StuI-digested pDAL866 to generate cosmid pDAL903. Cosmid pDAL878 is a CDI^-^ derivative of pDAL660Δ1-39 [2] in which the *cdiA-CT* ^EC93^ */cdiI* ^EC93^ toxin/immunity region has been deleted. Primers 1527/1663 were used to amplify a DNA fragment from strain DL6105 [5] and the resulting product was digested with SphI/AvrII and ligated to SphI/AvrII-digested cosmid pDAL660Δ1-39.

The chimeric CdiA^EC93^-CT^3937−2^ CDI system (cosmid pCH10323) was constructed in several steps. First, the kanamycin resistance cassette from plasmid pCH70 [28] was amplified with primers kan-Hind-for/kan-Eco-rev and ligated into HindIII/EcoRI-digested plasmid pKSS [29] to generate pKSS-Kan. Regions flanking the *cdiA-CT*_o1_^EC93^ sequence on cosmid pDAL879 [5] were then amplified with primer pairs EC93-Kpn-for/EC93-Hind-rev and EC93_o1_-Bam-for/EC93_o1_-Sac-rev. The PCR products were ligated sequentially into pKSS-Kan using KpnI/HindIII and BamHI/SacI restriction sites. The large KpnI/SacI fragment from the resulting plasmid (pCH10412) was gel-purified and electroporated together with cosmid pDAL879 into *E. coli* DY378 cells expressing the phage λ Red proteins as described [30]. Transformants containing recombined cosmids, which contain the kan^R^-pheS* cassette in place of the *cdiA-CT*_o1_^EC93^ toxin sequence, were selected on LB-agar supplemented with 50 µg/mL kanamycin. The resulting pCdiA-CT/pheS* cosmid was used for allelic exchange with *cdiA-CT* ^3937−2^/*cdiI* ^3937−2^ as follows. The *cdiA-CT* ^3937−2^/*cdiI* ^3937−2^ coding region was amplified with primers 3937-CT2-for/3937-cdiI2-rev. An upstream *cdiA* ^EC93^ homology region was amplified with primer 1527 and 3937CT2-chim-rev; and a downstream homology region amplified with primers 3937CT2-chim-for and 2368. The three PCR products were combined by overlapping extension-PCR (OE-PCR) [27] using oligonucleotides 1527/2368. The final product (100 ng) was electroporated together with 300 ng of cosmid pCdiA-CT/pheS* into *E. coli* DY378 and cells carrying the recombinant cosmid (pCH10323) were selected on YEG-agar (0.5% yeast extract, 1% NaCl, 0.4% glucose, 15 g agar/L) supplemented with 33 µg/mL chloramphenicol and 10 mM D/L-*p*-chlorophenylalanine.

The kanamycin-resistance cassette of pZS21-*bamA* (Kim et al., 2007) was replaced with a chloramphenicol acetyltransferase (*cat*) marker. The *cat* gene from *Salmonella enterica* TT23216 was amplified using oligonucleotides 2596/2597, digested with SacI/AatII and ligated to SacI/AatII-digested pZS21-*bamA* to generate plasmid pDAL950. The coding sequence for DsRed-Express2 was excised from plasmid DsRed-Express2 (Clontech) using NcoI/SpeI digestion, and the fragment subcloned into pTrc(DAS) [5] to generate plasmid pCH9817. Plasmid pDAL852 is a pBR322 derivative that expresses the *cdiI* ^3937−2^ gene from the *tet* promoter [1]. The identities of all plasmid and cosmid constructs were confirmed by DNA sequencing.

### Growth Competition Assays

Inhibitor cells carrying CDI cosmids [pWEB-TNC (mock CDI^-^), pDAL878 (mock CDI^-^), pDAL866 (CdiA^UPEC536^), pDAL903 (HA-CdiA^UPEC536^) or pCH10323 (CdiA^EC93^-CT^3937−2^)] were mixed with *E. coli* target cells (JCM158 Δ*wzb* or MC4100 Δ*wzb* derivatives) at various ratios and incubated in baffled flasks with shaking (250 rpm) at 37°C. Viable target cells were quantified as colony forming units (CFU) per mL by serial dilution onto LB agar plates supplemented with the appropriate antibiotic. For fluorescence microscopy of CDI co-cultures, target cells carried either plasmid pDAL672 or pCH9817, which express DsRed from the *lac* and *trc* promoters (respectively). *E. coli* DL4259 cells carrying pCH10323 were used as inhibitors for visualization of CdiA-CT^3937−2^ DNase activity during CDI. DL4259 contains the phage λ640-13 lysogen, which constitutively expresses *gfp-mut3* under control of the *papIB* promoter [31], [32].

### Antiserum Preparation and Whole-cell Immunoblotting

Polyclonal antiserum against residues Val3016– Ile3242 of CdiA^UPEC536^ was raised in rabbits (CoCalico Biologicals) [8]. Non-specific antibodies were removed by incubation with carbonyldiimidazole-activated agarose beads linked to soluble protein from *E. coli* strain CH2016 [33]. Briefly, protein-linked beads were resuspended in 0.5 mL of antiserum and the slurry mixed by rotation for 1 h at room temperature and an additional 8 h at 4°C. This process was repeated at least four times with fresh beads. Antiserum used to detect internal antigens via immunofluorescence microscopy was further purified by incubation with insoluble *E. coli* cell material. All adsorbed antisera were stored at 4°C with 0.2% sodium azide as preservative. For whole-cell immunoblotting, cells were grown to mid-log phase in TB medium, collected by centrifugation and fixed with 4% formaldehyde in phosphate buffered saline (PBS, pH 7.4) for 15 min. Fixation reactions were quenched with 100 mM glycine (pH 7.4), and the cells were permeabilized with 0.1% Triton X-100 and 0.1% sodium citrate in PBS for 15 min. Cells were incubated with antibodies anti CdiA-CT^UPEC536^ antiserum, mouse monoclonal anti-HA antibody (Covance), or mouse monoclonal anti-maltose binding protein (New England Biolabs)] in PBS supplemented with 1% bovine serum albumin (BSA). After washing with PBS, cells were incubated with goat anti-rabbit IRdye®800 or goat anti-mouse IRdye®680 secondary antibodies (Licor). Cells were washed three times in PBS, spotted onto nitrocellulose membranes, dried for 1 h and visualized with the Odyssey Infrared Imaging System (Licor).

### Immunofluorescence Microscopy

Competition co-cultures were collected and fixed with formaldehyde as described above. Where indicated, cells were treated with proteinase K (100 microgram/mL) for 30 min at 37°C prior to fixation. Cells mixtures were washed with PBS and resuspended in PBS supplemented with 1% BSA and purified rabbit anti-CdiA-CT antiserum or mouse monoclonal anti-HA antiserum (HA.11, Covance). After incubation for 30 min, cells were washed with 1% BSA in PBS and resuspended in 1% BSA in PBS supplemented with the appropriate fluorescent secondary antibody [goat anti*-*rabbit AlexaFluor®488, goat anti-mouse AlexaFluor®488, or goat anti-rabbit AlexaFluor®488 and goat anti-mouse AlexaFluor®568 secondary antibodies (Invitrogen)] for 30 min on ice. After washing, cells were resuspended in PBS supplemented with 0.2% sodium azide, spotted onto poly-D-lysine coated glass slides and sealed with FluoroGel II (Electron Microscope Sciences). Samples were visualized using an Olympus BX51 microscope, Omega filters XF116-2 (for EGFP) and XF103-2 (for Ds-Red) and an Optronics (Goleta, CA) camera (gain = 16 and 500 ms fluorescence exposure). Overlaid images were adjusted for brightness and contrast using Adobe Photoshop CS2. GFP images were layered over Ds-Red images at 75% opacity. GFP signal brightness was adjusted to –30 and contrast adjusted to +30, images flattened, and curves adjusted to 110 input and 250 output. For visualization of *in vivo* DNase activity, fixed cells were stained with SlowFade gold solution with DAPI (Invitrogen) supplemented with 0.1% Triton X-100. Camera settings were as follows: brightfield imaging: 30 ms exposure, gain = 2; DAPI staining: 200 ms exposure, gain = 2; DsRed: 1 s exposure, gain = 10; and GFP: 1 s exposure, gain = 10. Images were processed using the GIMP image enhancement software and altered for contrast enhancement opacity for image overlays.

For detection of internal antigens, cell fixation and permeabilization was conducted as described [34] with the following modifications. Where indicated, cells were treated with 100 µg/mL proteinase K at 37°C for 30 min prior to fixation. Cells were fixed in 2.6% formaldehyde, 0.04% glutaraldehyde in 32 mM sodium phosphate (pH 7.4) for 10 min at room temperature and 50 min on ice. After washing with PBS, cells were resuspended in 50 mM glucose, 20 mM Tris-Cl (pH 7.5), 10 mM EDTA supplemented with 0.02 mg/mL lysozyme. The cell suspension was spotted onto a poly-D-lysine-coated slide and incubated for 10 min. Unbound cells were removed with three water rinses and the slides air-dried for 45 min. After rehydration in PBS for 4 min, samples were incubated with 1% Blocking Reagent (Invitrogen) in PBS, then stained with either rabbit anti-CdiA-CT or mouse anti-HA antibodies. After washing, cells were incubated with goat anti-rabbit IgG or goat anti-mouse IgG secondary antibodies conjugated to horseradish peroxidase. Samples were washed with PBS and incubated in amplification buffer (Invitrogen) containing 0.0015% hydrogen peroxide and 1∶1000 tyramide AlexaFluor®488 (Invitrogen) for 5 min. Slides were washed with PBS, sealed with a drop of FluoroGel II (Electron Microscope Sciences) and coverslip, then analyzed with fluorescence microscopy as described above for surface antigens.

## Supporting Information

Figure S1 **Immunodetection of cell-surface and internal antigens.** A) Whole-cell immunoblot for maltose-binding protein (MBP). Wild-type *E. coli malE^+^* (MBP^+^) and Δ*malE* (MBP^-^) cells were incubated with anti-MBP antibodies and spotted onto nitrocellulose membrane for fluorescence imaging as described in Methods. Where indicated (+), samples were treated with proteinase K (proK) to remove cell-surface antigens, and/or Triton X-100 (PERM) to permeabilize the cells. B) Immunofluorescence microscopy of MBP. Wild-type *E. coli malE^+^* (MBP+) and Δ*malE* (MBP^-^) cells were analyzed by fluorescence microscopy using anti-MBP antibodies. Where indicated (+), samples were treated with proteinase K (proK) to remove cell-surface antigens, and/or lysozyme and EDTA (PERM) to permeabilize the cells. Together, these control experiments demonstrate that internal/periplasmic antigens are not degraded by proteinase K treatment and that internal antigens are only detected when cells have been permeabilized.
(TIF)

Figure S2 **HA-CdiA^UPEC536^ is fully functional in CDI.** Inhibitor *E. coli* cells carrying cosmids pDAL866 (CdiA^UPEC536^), pDAL903 (HA-CdiA^UPEC536^), or pWEB-TNC (CDI^-^ vector control) were co-cultured with target cells (JCM158 Δ*wzb::kan*). Viable target cells were quantified as colony forming units (CFU) per mL by plating co-cultures onto LB agar supplemented with kanamycin. Values represent the average ± SEM for at least two independent experiments.
(TIFF)

Table S1 **Oligonucleotides used in this study.**
(DOCX)
