# Supplementary figures and images for: Delivery of CdiA Nuclease Toxins into Target Cells during Contact-Dependent Growth Inhibition
Source: PLoS One. 2013 Feb 28;8(2):e57609. doi: 10.1371/journal.pone.0057609 (PMC3585180; doi:10.1371/journal.pone.0057609)

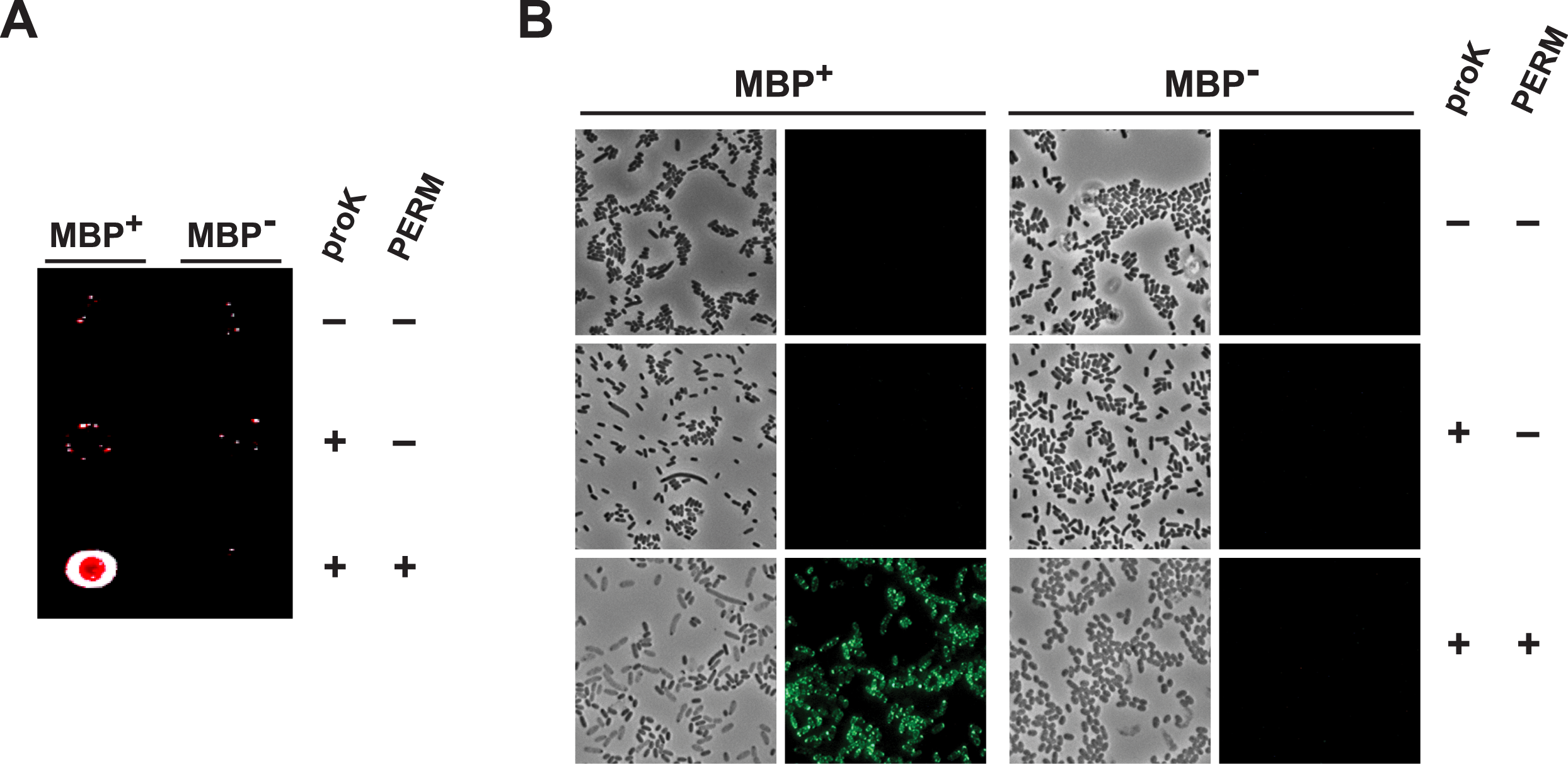

Supplement: Figure S1 — Immunodetection of cell-surface and internal antigens. A) Whole-cell immunoblot for maltose-binding protein (MBP). Wild-type E. coli malE+ (MBP+) and ΔmalE (MBP-) cells were incubated with anti-MBP antibodies and spotted onto nitrocellulose membrane for fluorescence imaging as described in Methods. Where indicated (+), samples were treated with proteinase K (proK) to remove cell-surface antigens, and/or Triton X-100 (PERM) to permeabilize the cells. B) Immunofluorescence microscopy of MBP. Wild-type E. coli malE+ (MBP+) and ΔmalE (MBP-) cells were analyzed by fluorescence microscopy using anti-MBP antibodies. Where indicated (+), samples were treated with proteinase K (proK) to remove cell-surface antigens, and/or lysozyme and EDTA (PERM) to permeabilize the cells. Together, these control experiments demonstrate that internal/periplasmic antigens are not degraded by proteinase K treatment and that internal antigens are only detected when cells have been permeabilized. (TIF) [file pone.0057609.s001.tif]

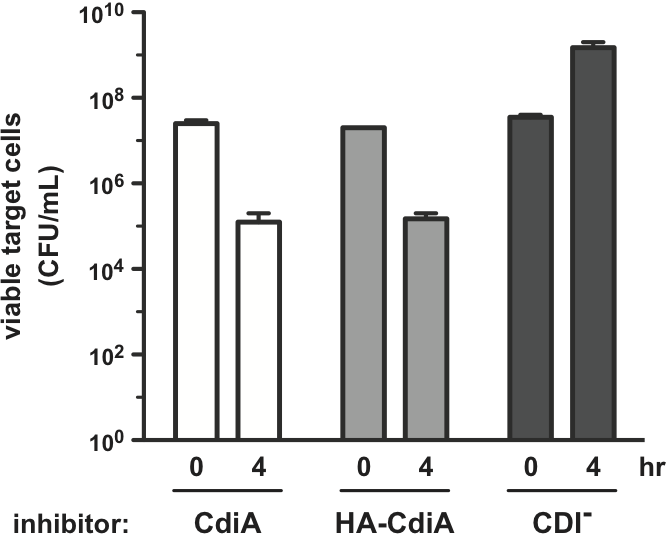

Supplement: Figure S2 — HA-CdiAUPEC536 is fully functional in CDI. Inhibitor E. coli cells carrying cosmids pDAL866 (CdiAUPEC536), pDAL903 (HA-CdiAUPEC536), or pWEB-TNC (CDI- vector control) were co-cultured with target cells (JCM158 Δwzb::kan). Viable target cells were quantified as colony forming units (CFU) per mL by plating co-cultures onto LB agar supplemented with kanamycin. Values represent the average ± SEM for at least two independent experiments. (TIFF) [file pone.0057609.s002.tif]
