# Supplementary material for: Delivery of CdiA Nuclease Toxins into Target Cells during Contact-Dependent Growth Inhibition
Source: PLoS One. 2013 Feb 28;8(2):e57609. doi: 10.1371/journal.pone.0057609 (PMC3585180; doi:10.1371/journal.pone.0057609)
Supplement: Table S1 — Oligonucleotides used in this study. (DOCX) [file pone.0057609.s003.docx]

**Table S1. Oligonucleotides used in this study.**

| **Oligonucleotide** | **Sequence** |
| --- | --- |
| 1527 | 5´ - GAA CAT CCT GGC ATG AGC G |
| 1663 | 5´ - GGT CTG GTG TCT AAC CTT TGG G |
| 2051 | 5´ - CAT GTT TCC GGT TCG TTC ACT G |
| 2052 | 5´ - GCT GTT GCG GAT GTC CGG TAT ATC |
| 2064 | 5´ - AGC GTA ATC TGG AAC ATC GTA TGG GTA GGC CCC CAC AGC CGG TAA C |
| 2065 | 5´ - TAC CCA TAC GAT GTT CCA GAT TAC GCT GTC ATC ACC CCA CAA AAC GGG |
| 2358 | 5´ - GTT GGT AGT GGT GGT GCT G |
| 2596 | 5´ - CAA CAA GAC GTC CAC CAA ACA CCC CCC AAA AC |
| 2597 | 5´ - CAA CAA GAG CTC CAC ACA ACC ACA CCA CAC CA |
| kan-Hind-for | 5´ - TAG AAG CTT CAA GAT CCC CTC ACG |
| kan-Eco-rev | 5´ - CCA GAA TTC CGC TCA GAA GAA CTC G |
| EC93-Kpn-for | 5´ - TAC GGT ACC GGC AGT ACG CCG CAG ATG |
| EC93-Hind-rev | 5´ - ATC AAG CTT AGC GAG TTA TTC TCA AC |
| EC93_o1_-Bam-for | 5´ - ATA AAC TTG GAT CCG CAA TAA AGG |
| EC93_o1_-Sac-rev | 5´ - TTT GAG CTC AGA ATA TCT ATT TAG |
| 3937-CT2-for | 5´ - TCG CTG GGG GAT ATT GCG CAG GCG C |
| 3937-cdiI2-rev | 5´ - TTA CTG CAT TAG TGT CAC TCT ACA TAG |
| 3937CT2-chim-rev | 5´ - GCG CCT GCG CAA TAT CCC CCA GCG AAT TAT TCT CAA CCG AGT TCC TAC CTG |
| 3937CT2-chim-for | 5´ - CTA TGT AGA GTG ACA CTA ATG CAG TAA CCC AAA GGT TAG ACA CCA GAC C |
|  |  |
